# The Role of Extracellular Vesicles in Viral Infection and Transmission

**DOI:** 10.3390/vaccines7030102

**Published:** 2019-08-28

**Authors:** Lorena Urbanelli, Sandra Buratta, Brunella Tancini, Krizia Sagini, Federica Delo, Serena Porcellati, Carla Emiliani

**Affiliations:** 1 Department of Chemistry, Biology and Biotechnology, University of Perugia, Via del Giochetto, 06123 Perugia, Italy; 2 Centro di Eccellenza sui Materiali Innovativi Nanostrutturati (CEMIN), University of Perugia, Via del Giochetto, 06123 Perugia, Italy

**Keywords:** extracellular vesicles, exosomes, mechanisms of viral spreading, Human Immunodeficiency Virus (HIV), Epstein-Barr virus (EBV), HCV

## Abstract

Extracellular vesicles (EVs) have been found to be released by any type of cell and can be retrieved in every circulating body fluid, namely blood (plasma, serum), saliva, milk, and urine. EVs were initially considered a cellular garbage disposal tool, but later it became evident that they are involved in intercellular signaling. There is evidence that viruses can use EV endocytic routes to enter uninfected cells and hijack the EV secretory pathway to exit infected cells, thus illustrating that EVs and viruses share common cell entry and biogenesis mechanisms. Moreover, EVs play a role in immune response against viral pathogens. EVs incorporate and spread both viral and host factors, thereby prompting or inhibiting immune responses towards them via a multiplicity of mechanisms. The involvement of EVs in immune responses, and their potential use as agents modulating viral infection, will be examined. Although further studies are needed, the engineering of EVs could package viral elements or host factors selected for their immunostimulatory properties, to be used as vaccines or tolerogenic tools in autoimmune diseases.

## 1. Introduction

Extracellular vesicles (EVs) have gained considerable attention in the last two decades. They were initially discovered by Johnstone as particles released by platelets during their differentiation [1]. For this reason, they were considered a tool that allows cells to dispose extracellularly unnecessary material. However, as soon as further studies on their biochemical characterization were carried out, it became known that EVs represent an additional method of signal transduction. For example, proteomic analysis rapidly identified the presence of Major Histocompatibility Complex (MHC) class I and II molecules in EVs, clearly indicating their involvement in antigen presentation [2,3]. EVs are membrane surrounded structures released in the extracellular milieu by every type of cell. Therefore, they have been retrieved in every fluid of the body, namely blood, urine, saliva, milk, cerebrospinal, amniotic, and ascitic fluid [4,5,6]. From a structural point of view, EVs are characterized by small dimensions and heterogeneity. They are classified in three main groups: exosomes, microvesicles, and apoptotic bodies. Exosomes originate from the inward budding of the late endosomal membrane, which produces small intraluminal vesicles (ILVs). This type of late endosome is called a Multi Vesicular Body (MVB), and, upon exocytosis, ILVs are released extracellularly, taking the name “exosomes”. On the other hand, microvesicles originate from the outward budding of the plasma membrane and are released as soon as they are produced. Exosomes and microvesicles have different average sizes, as exosomes are reported to range from 30 to 120 nm and microvesicles from 100 to 1000 nm. In addition, it has been recently evidenced that vesicle populations sharing the same size can have different densities [7,8]. Due to the drawbacks and pitfalls of current separation methods, which are mostly based on size and density, it is difficult to obtain “pure” exosome or microvesicle preparations. Consequently, it is currently recommended to use the term small EVs (enriched in exosomes) for vesicles prepared by ultracentrifugation at high speeds (100,000 g), and medium/large EVs (enriched in microvesicles) for vesicles prepared by centrifugation at lower speeds (10,000 g). Finally, cells undergoing apoptosis release “apoptotic bodies”, i.e., EVs of a heterogeneous size (50–5000 nm), produced by cells undergoing apoptosis [9]. Although less commonly investigated than exosomes and microvesicles, apoptotic bodies are a heterogenous population generated by membrane blebbing and membrane protrusion, functionally involved in the clearance of apoptotic material and the modulation of immune response [10].

The content of EVs has been extensively investigated. EVs contain proteins, lipids, nucleic acids, and other metabolites. The biochemical composition of EVs is different than their releasing cell. Therefore, it has been suggested that most EV biochemical components are actively packaged within EVs by specific molecular mechanisms, rather than passively included. However, EV content is also reminiscent of the releasing cell, i.e., EVs released by endothelial cells contain endothelial cell-specific proteins, although their types and levels differ from the parental cell [11,12,13]. For this reason, in principle, it is possible to identify a subpopulation of EVs deriving from a specific tissue and/or cell type in a heterogenous population of EVs present in bodily fluids, such as blood, thereby allowing the development of specific biomarkers from circulating EVs. However, this goal is far from being reached.

The protein content of EVs has been extensively investigated. Small EVs enriched in exosomes are characterized by the presence of proteins of endosomal origin involved in exosome biogenesis, such as Alix and Tsg101. Other proteins, which are commonly retrieved and consequently used as biomarkers, are tetraspanins such as CD63, CD9, and CD81, which have also been involved in exosome biogenesis, heat shock proteins (HSPs) such as HSP70, and small GTPases. Finally, the presence of proteins modulating immune response has been also often reported (Fas Ligand (FasL), Transforming Growth Factor beta (TGF-β), Tumor Necrosis factor alfa (TNF-α)). Although these proteins are the most well-known biomarkers for exosomes, they can also be retrieved at different level in other vesicles, such as large/medium EVs enriched in microvesicles [7,14,15]. In addition, microvesicles are usually enriched with cell surface proteins, such as receptors, integrins, and selectins [16].

The lipid composition of small EVs enriched in exosomes is different from that of the releasing cell, although it is related to the cell type. As for structural lipids, small EVs are enriched in cholesterol and sphingolipids, indicating that their membrane composition resembles that of lipid rafts [17,18]. Besides structural lipids, EVs contain also bioactive lipids, such as prostaglandins, and lysophospholipids [19,20]. The lipid composition of large/medium EVs, enriched in microvesicles, has been much less frequently investigated. However, a few studies have provided evidence that microvesicles are enriched in ceramides and sphingomyelins and are characterized by a higher level of polyunsaturated species compared to exosomes [21,22].

EVs have also aroused considerable interest because they carry different types of nucleic acids, (namely miRNAs [23], but also, as more recently revealed, lncRNA and circRNA) [24]. Interestingly, one of the most relevant proofs demonstrating miRNA delivery into recipient cells via EVs was the detection of viral miRNA released from the Epstein–Barr virus (EBV)-infected cells within non-infected cells [25]. The presence of miRNAs within EVs isolated from circulating body fluids has stimulated a large amount of studies aiming to investigate the diagnostic and prognostic potential of EVs. In fact, the amount and type of miRNAs that can be retrieved within EVs are different from the cellular miRNA content, thereby implicating a mechanism of active miRNA packaging within EVs [26]. Similar findings have been described for lncRNA [27]. Further, the vesicular miRNA content is affected by the pathological state of the cells. For example, infected cells release not only viral miRNAs, but also infection associated miRNAs. The nucleic acid content of EVs is also relevant for additional reasons. In 2007, Valadi et al. [28] published a seminal paper reporting not only the presence of mRNA within exosomes, but also its ability to be transferred and expressed in recipient cells, thereby affecting their expression profile. This was the first report demonstrating that the nucleic acids could be transferred horizontally from cell to cell, directly influencing the expression profile.

The presence of DNA within EVs has also been described. Indeed, the discovery of mtDNA, ssDNA, and dsDNA has been reported, and these findings have reinforced the role of EVs as a means of horizontal gene transfer. Most mtDNA is adsorbed outside of vesicles [29]. Nevertheless, it has been recently shown that EVs from Kaposi’s sarcoma associated herpesvirus (KSHV)-infected cells stimulate an antiviral immune response through mtDNA [30]. Large EVs carry most of the tumour dsDNA circulating in prostate cancer patient plasma [31]. The presence of oncogenic virus dsDNA in EVs may be relevant for oncogenic transformation. In fact, it has been shown that infected cells release EVs containing viral dsDNA that regulate innate immune responses to HBV infection [32].

From a functional point of view, it is now recognized that EVs play a prominent role in immune system development and function, during both adaptive and innate immune responses [33]. For adaptive immune response, initial proteome analysis of the EVs provided evidence that EVs carry MHC class I [34] and II [3] molecules. Then, it was found that EVs released by Antigen Presenting Cells (APC) are involved in the direct antigen presentation to T cells via the association of antigenic peptides with MHC class I and II receptors and, therefore, can stimulate CD4^+^ and CD8^+^ T cells, respectively [35]. Besides the direct antigen presentation by professional APCs to T cells, EVs released by any cell type can be captured by APCs and then present antigens in an indirect manner [36]. This mechanism of indirect antigen presentation by EVs is very relevant when cells are infected, as EVs released by infected cells can disseminate pathogen antigens [37]. Finally, this mechanism is also a relevant feature of anti-tumor immune responses, as the presence of tumor antigen peptides associated with MHC complexes in EVs released from cancer cells has been shown to elicit a specific cytotoxic T lymphocyte response [38]. These findings have prompted the exploitation of dendritic cell (DC)-derived exosomes as a novel cell-free vaccine to eradicate murine tumors [2], leading to clinical trials to validate their use for cancer therapy [39]. Nevertheless, it later became clear that EVs also possess immunosuppressive functions, as tumor-derived EVs can directly suppress the activity of effector T cells and the function of Natural Killer (NK) cells [40]. It is now clear that the separation protocols used, and the culture conditions of the releasing cells, greatly affect the outcome of the functional assays. Further studies are needed to define the immunostimulatory and immunosuppressive function of EVs.

In this context, we are going to review the current knowledge on the role of EVs in viral infection and the role of their biochemical content in prompting viral spread and modulating immune response, by either favoring infections or limiting them.

## 2. Extracellular Vesicles

### 2.1. Biogenesis

The biogenesis of EVs is diversified. As already mentioned, exosomes originate from the inward budding of the late endosomal membrane, producing ILVs with small dimensions (30–120 nm). The pathways leading to the production of ILVs can be classified in Endosomal Sorting Complex Required for Transport (ESCRT)-dependent and independent mechanisms.

ESCRT is a type of molecular machinery involved in membrane remodeling and scission in many processes, including cytokinesis [41]. The involvement of the ESCRT system in exosome biogenesis was postulated upon the discovery that many ESCRT proteins are retrieved in exosomes. In fact, the above mentioned Tsg101 (a ESCRT-I component) and Alix (an accessory protein) are among the most widely used markers for exosomes [14,15]. ESCRT machinery is composed of four protein complexes (ESCRT-0, -I, -II, -III) alongside other proteins, such as Alix, that play a key role. The four multimolecular complexes act sequentially in a multistep process, leading first to cargo recognition (ESCRT-0) and then to cargo recruitment and membrane invagination (ESCRT-0, I and -II), vesicle maturation and neck constriction (ESCRT-III), and finally to inward membrane scission, which involves the vacuolar ATPase Vps4 [42]. More recently, an alternative ESCRT-dependent pathway has also been described; this pathway relies on the ESCRT accessory protein Alix. In this case, evidence has been provided that syndecan, a heparan sulphate, is internalized from the cell surface to the endosomes, and interacts on the cytosolic surface with syntenin, which in turn binds to Alix and other ESCRT components promoting inward membrane invagination [43].

However, studies inactivating ESCRT components have provided evidence that exosomes can be produced by additional mechanisms [44], and, specifically, two ESCRT-independent mechanisms have been reported. In one case, evidence has been provided that lipids are not only structural constituents but also play a role in vesicle biogenesis [45]. In fact, neutral sphingomyelinase (nSMase) is responsible for the production of ceramide on the surface of the endosomal membrane. This lipid has a conic shape that promotes a spontaneous negative curvature, leading to the generation of ILVs. In another case, the ESCRT-independent mechanism was shown to rely on tetraspanins. Like the previously mentioned ESCRT components, Tsg101 and Alix, tetraspanins are among the best characterized markers for EV preparation. A few studies have provided evidence that the sorting of specific proteins into exosomes is dependent on tetraspanin clustering on the surfaces of late endosomes/MVBs. Indeed, the PreMelanosome protein (PMEL) is sorted into ILVs through a process involving CD63 tetraspanin [46,47].

Nevertheless, it is worth recalling that ESCRT-independent and ESCRT-dependent pathways not only represent alternative routes but also act, at least in some cases, synergically. Indeed, it has been reported that ESCRT and the ceramide pathway may function together for the secretion of phospholipid scramblase 3 within exosomes [48], and ceramide and tetraspanin pathways can act together in the disposal of β-catenin via exosomes [49]. Further evidence of multiple mechanisms in the secretion of exosomes is given by the fact that the inhibition of a single pathway (for example, the inhibition of ceramide synthesis using the nSMAse inhibitor) does not suppress exosome release [50].

In the case of microvesicles shedding from the plasma membrane, the microvesicles are released as soon as they are produced, but their biosynthesis has been much less commonly investigated. The first event of their biogenesis is the accumulation of cargo on the cytosolic face of the plasma membrane. Then, the outward membrane curvature begins, accompanied by specific and localized changes in plasma membrane’s lipid and protein composition. Despite the different topological localization, part of the endosomal machinery used for exosome biogenesis is also used for microvesicle formation. Indeed, ESCRT components, such as Tsg101, have been demonstrated to also be involved in microvesicle formation [51]. Another important protein involved in microvesicle formation is the small GTPse Arf6 and other small GTPases of the Rho family, which are involved in the contraction of actin beneath the plasma membrane [52].

**Figure 1:**
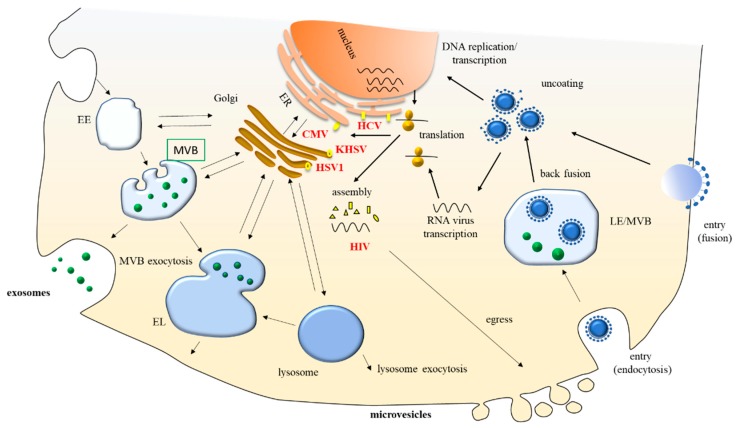
Common routes in virus life cycle and Extracellular Vesicle (EV) biogenesis and release. EV biogenesis requires certain components and takes place in cellular compartments that are in close communication with the assembly and release sites of viruses, despite differences in the biology of each virus, allowing the formation of vesicles containing viral elements. EE, early endosomes; MVB, Multivesicular Bodies; LE, Late Endosome; EL, Endolysosome; ER, Endoplasmic Reticulum; KSHV, Kaposi’s sarcoma-associated herpesvirus; EBV, Epstein–Barr virus; CMV, Cytomegalovirus; HSV1, Herpes simplex virus; HIV, Human immunodeficiency virus; HCV, Hepatitis C virus.

The ESCRT complex is pivotal in the biogenesis of both small and medium/large EVs, and several viruses have been shown to interact with ESCRT components, suggesting converging pathways between virus life cycle and EV biogenesis or release. Viruses attach to a specific receptor on the cell membrane through capsid or envelope proteins. Then, most animal viruses enter through endocytosis, although some enveloped viruses enter the cell when the viral envelope fuses directly with the cell membrane. Inside the cell, the viral nucleic acid becomes available for replication and/or transcription, but the cell compartment in which the replication and assembly processes take place depends on the virus. Once assembled, viruses can be either released when the host cell dies or leave infected cells by budding through the membrane. In the case of membrane budding, viruses egress directly from the plasma membrane (e.g., Human Immunodeficiency Virus, HIV)) or into an exocytic host pathway (e.g., Hepatitis C virus, HCV). HIV hijacks the ESCRT pathway using late assembly domains within its structural protein, Gag, which can recruit ESCRT-I components and Alix [53], whereas Herpesviruses typically acquire their final envelopes in various cytoplasmic compartments, such as the trans-Golgi network (TGN) and endosomes prior to their secretion into the extracellular space, interacting with ESCRT components [54,55] (Figure 1). Indeed, the initial discovery of EVs has rapidly led to the hypothesis, formulated by Stephen J Gould, that viruses spread through immune cells, exploiting EVs like a “Trojan horse”. This hypothesis was suggested by the finding that HIV could exploit exosomes released from the late endosomal compartment of mature DC to trans-infect CD4^+^ T cells in lymph nodes [56]. Further functional studies on EVs released by infected cells have demonstrated that infected cells release not only infective virions, which are able to spread the infection, but also a variety of non-replicative particles, which are difficult to classify, as they could be considered either defective virus particles or EVs containing viral elements, such as viral proteins and nucleic acids. Independently of their classification, these EVs can modulate the host’s immune response. However, it is difficult to assign a specific function to EVs released by infected cells, because it is very difficult to fully separate replication-competent virions and EVs, as there is a continuum between EVs without viral elements and replication-competent virions, which share similar size, density and biochemical content, as many viral proteins are present both in EVs and virions, although to a different extent [57]. As the current separation methods do not allow to separate different types of vesicles and are not fully adequate to separate EVs from viruses and/or defective viruses, it may be possible that some functional studies aimed at addressing the infection ability of EVs containing viral elements may be affected by the co-purification of viral particles, either defective or not.

### 2.2. Uptake

EVs have been reported to be uptaken by recipient cells through a multiplicity of mechanisms, although many details are unclear. In fact, as EVs are heterogenous, different type of vesicles may be internalized by different types of mechanisms, so the delivery route of EV cargo may be dependent on the type of EV released. Basically, EVs can transmit information to a recipient cell in two ways: by interacting with specific receptors and/or adhesion molecules on the cell membrane or by fusing with the plasma membrane, thereby releasing their content within the cell.

Many proteins localized on the cell surface have been proposed to function as receptors and/or adhesion molecules, such as integrins and proteoglycans. It has been shown that EVs can bind to proteoglycans, such as heparan sulphate, on the cell surface [58], as well as integrins [59], lectins [60], and tetraspanins [61]. Nevertheless, there is no evidence that EV internalization can be completely arrested by blocking one of these receptors. In fact, it has been shown that antibodies that mask the binding sites of CD11a or its ligand Intercellular Adhesion Molecule (ICAM)-1 can reduce the DC uptake of EVs but cannot abolish it [62]. Further, in this model, the treatment of recipient cell with antibodies against the tetraspanins CD81 or CD9 can reduce the uptake of EVs by DCs, but again, it cannot abolish the uptake [62].

Once bound to the cell surface, EVs are internalized by endocytosis through various mechanisms, both clathrin-dependent [63] and independent [64]. The implication of clathrin-mediated endocytosis in the uptake of EVs has been determined by the finding that the inhibition of dynamin2 (a GTPase required for clathrin-mediated endocytosis in phagocytic cells) prevents almost all EV internalization activity [65]. For clathrin-independent mechanisms, EVs can be internalized by caveolin-dependent endocytosis. Caveolae are plasma membrane sub-domains of glycolipid rafts enriched in cholesterol, sphingolipids, and caveolins. Dynamin 2 participates in the assembly and expansion of caveolar vesicles. Blocking dynamin 2 leads to significantly reduced internalization of exosomes [66] or microvesicles [67], suggesting a role for caveolae-mediated endocytosis in vesicular uptake. However, as just mentioned above, dynamin2 is also required for clathrin-mediated endocytosis, so it is not possible to rule out a role for clathrin-coated vesicles in these experiments. These results demonstrate that it is very difficult to provide evidence that only one pathway is involved in EV internalization, indicating that more than one pathway may be involved at the same time in the same cell.

Other endocytic mechanisms have been proposed to be involved in EV uptake, such as macropinocytosis. This process allows eukaryotic cells ingest extracellular liquid, including macromolecules that are dissolved in the liquid. It has been proposed that when EVs are present in the extracellular liquid, they can be internalized via macropinocytosis. Indeed, during micropinocytosis, the plasma membrane protrudes from the cell’s surface and surrounds the extracellular liquid, producing a vacuole that undergoes acidification. This vacuole can include EVs that are present in the extracellular liquid. This process relies on cytoskeleton proteins, such as actin, on small GTPases (such as Rac1, and on the Na^+^/H^+^ exchanger) to regulate its contractions. Thus, when vacuole acidification is prevented by Na^+^/H^+^ exchanger inhibitors or by alkalinizing drugs, such as bafilomycin A and chloroquine, EV uptake is inhibited [68]. Nevertheless, the use of macropinocytosis inhibitors to block EV uptake has not always been successful, so it has been suggested that the use of macropinocytosis as a mechanism for EV internalization could be dependent on cell type or may represent one of many pathways [65].

Although phagocytosis is a receptor-mediated mechanism that allows the internalization of large particles by specialized cell types, it has also been involved in EV internalization by macrophages [60]. The use of inhibitors, such as wortmannin and LY294002, has provided evidence that functional phosphatidyl inositol 3 kinases are important for EV uptake by phagocytosis [65], together with the interaction of phosphatidylserine (PS) present on the external leaflet on the EV membrane with a PS receptor, such as translocase of the inner membrane (TIM)-4 on the macrophage’s surface [62].

To completely describe the mechanisms behind EV internalization, studies have demonstrated that EV uptake could also depend on lipid rafts, independently from clathrin or caveolin. Lipid rafts are enriched in glycosphingolipid and cholesterol, and studies have demonstrated that decreasing glycosphingolipid biosynthesis using specific inhibitors reduced EV uptake [69]. Thus, EV uptake is prevented following the pre-treatment of recipient cells with cholesterol reducing agents, such as methyl-β-cyclodextrin (MβCD) [70].

Apart from endocytic mechanisms, the other possible entry mechanism is via direct fusion of the EV membrane with the cell plasma membrane. Indeed, the miRNA and mRNA content of EVs must reach the cytosol to finalize its function and direct fusion with plasma membrane may represent the most rapid method. Evidence in favor of a direct fusion of EVs with plasma membrane has been provided. This evidence shows the delivery of miRNAs and luciferin to the cytosol of the recipient cell [71]. However, the quantitative contribution of this pathway compared to the other endocytic mechanisms is not clear.

Finally, it is worth recalling that the delivery of EV content within the cell is a process that must take place even when internalization occurs via endocytosis, micropinocytosis, or phagocytosis, otherwise EVs would remain confined to endosomes. The mechanisms of endosomal escape, which implicate the fusion of the EV membrane with the endosome membrane, are not clear and have not been extensively investigated. However, it has been suggested that the endosome’s acidic pH could play a role in stimulating EV membrane fusion [71].

Notably, it has also been proposed that viruses may have adopted existing EV-mediated communication pathways for their infection strategies [72], thus exploiting common pathways in EV and viral uptake. A notable example is the recent finding that many enveloped viruses utilize PS receptors as part of their targeting and entry strategies [73,74]. The presence of PS on the surfaces of EVs is a common finding in different studies, and it has been hypothesized that it might also be relevant for EV entry [75]. However, not all EVs have been demonstrated to expose PS, so it is presumable that not all EVs release their content in the same way.

Many aspects of EV uptake must still be elucidated, but it is clear that most viruses enter cells by endocytosis. Therefore, endocytic mechanisms represent a common crossroad between EVs and virus entry. Conversely, the endosomal compartment represents a common intersection between EV/virus entry and EV/virus assembly (Figure 1). In fact, for RNA viruses like HCV, the fusion with the endosomal membrane upon endocytosis, and the delivery of the viral nucleocapsid into the cytoplasm, are distinct events. During these processes, virus particles fuse preferentially with exosomes, which then depend on the back-fusion of the exosomes with the late endosomal membrane to deliver the viral nucleocapsid into the cytoplasm [76].

## 3. Extracellular Vesicles in Viral Transmission

The involvement of EVs in viral spread and infection has become evident by two converging lines of research. On the one hand, it has emerged that EVs and viruses share common features in their size, structure, biogenesis (as for example, both EVs and viruses use the ESCRT complex for their biosynthesis), and uptake. On the other hand, several studies investigating EVs released from infected cells showed that these EVs contain viral components, which include proteins, but also genetic material, such as viral miRNAs [57]. This viral content has been demonstrated to be either beneficial or detrimental to the host’s immune response, at least in a few experimental models, further complicating the picture. In addition, evidence has also shown that infection modifies the incorporation of cellular proteins and nucleic acids in EVs. Thus, independent of viral element content, infected cells produce modified EVs. These “modified EVs” can also affect the host’s immune response, with respect to their counterparts released by non-infected cells. Finally, EVs are vehicles for the en bloc delivery of viral particles, allowing multiple genomes to collectively infect the same cell, often resulting in enhanced infectivity. Indeed, they join structures such as aggregates of virions and virion-containing protein aggregates to increase the multiplicity of infections, exploiting different entry routes and maintaining genetic diversity [77].

### 3.1. The Role of EVs in Spreading Infection

EVs can deliver much material between cells, for example, proteins (both transmembrane and soluble); bioactive lipids, such as prostaglandins and lysophosphatidylcholine; nucleic acids, namely miRNAs; and metabolites. All these components have an impact on recipient cells and on the immune response. In this section we are going to introduce studies that show the impact of different biomolecules delivered via EVs on antiviral response. Most of these studies were carried out on HIV and EBV, but relevant results have also been obtained for other viruses and will be considered.

EVs can deliver proteins that make the cell more susceptible to infection. HIV proteins have been demonstrated to be vehiculated in recipient cells via EVs and to induce the spreading of infection by rendering cells more susceptible to infection. One of the best-known and most investigated examples is the HIV protein Nef. HIV Nef is released and vehiculated via EVs [78,79]. Latent HIV-1 can be activated by exosomes released from cells infected with either replication-competent or defective HIV-1, but this effect was no more detectable when exosomes from cells infected with HIV-1 strains (either Nef-deleted or Nef-defective) were used, suggesting that vesicular Nef renders cells more susceptible to HIV infection [80]. In addition, vesicular Nef can induce cell senescence or cell death in bystander CD4^+^ T lymphocytes [81,82]. Nef can decrease the incorporation of CD4 into T cell EVs, thereby preventing the binding of virions to vesicular CD4 and increasing the amount of circulating virus available to infect the cell [83]. Further, Nef packaged in macrophage-released EVs can induce degradation within the lysosomal compartment of CD4, thus making cells less susceptible to cytotoxic immune responses [84]. Finally, Nef transferred via EVs can influence the adaptive immune response acting not only on T cells, but also on B cells, thereby facilitating the evasion of the humoral immune response by suppressing IgG and IgA production in B cells [85].

Another mechanism that can make cells more susceptible to infection is the delivery, via EVs, of viral receptors to cells that are devoid of these receptors, thereby allowing these cells to be infected. Investigations on HIV have provided a few examples of this mechanism. Indeed, platelet- and megakaryocyte-derived EVs can transfer the HIV co-receptors C-X-C chemokine receptor type 4 (CXCR4) to the CXCR4-null target cell [86] and, similarly, C-C chemokine receptor type 5 (CCR5) can be released via EVs from CCR5+ Chinese hamster ovary cells and peripheral blood mononuclear cells, thus transferring the receptor to the CCR5 null cell [87]. This mechanism renders cells ready to be infected by HIV in vitro, but the in vivo implications of this extended tropism remain unclear.

Nucleic acids contained within EVs can also induce the spreading of infection by improving and sustaining the production of a virus in infected cells, and examples of this mechanism have been described for HIV infection. In fact, EVs released from HIV-infected cells contain a peculiar nucleic acid, i.e., a TAR (transactivation response element) RNA. TAR has a stem-loop structure located at the 5′ ends of HIV transcripts, which, in infected cells, can be recognized and bound by the Tat protein, thus increasing the production of viral RNA. Interestingly, the full-length TAR RNA can be processed into miRNAs that silenced a Bcl-2 interacting protein. This induces an increase in apoptosis resistance, which can prolong the virus’s production [88,89].

HIV infection is not the only infection model that has been investigated so far. Other examples of EVs released from infected cells affecting antiviral response come from DNA viruses, such as herpesviruses, namely EBV. As for HIV, EBV-infected cells release EVs that incorporate viral proteins, such as Latent Membrane Protein 1 (LMP-1) [90]. LMP1 mimics CD40 signaling and induces the proliferation of B lymphocytes and T cell-independent class-switch recombination [91]. The presence of LMP-1 in the exosomes is regulated by association with the CD63 tetraspanin and has been shown to limit constitutive NF-kappa B activation [92,93]. Additional signaling pathways, such as fibroblast growth factor (FGF)2, are affected by the increased packaging of FGF2 in exosomes induced by LMP-1 [94]. When exosomes released from EBV-infected cells are internalized by recipient cells, their LMP-1 content can up-regulate adhesion molecules, such as ICAM-1, in recipient cells [66]. Besides LMP-1’s role in promoting infectivity, the EV protein’s composition was shown to be affected by EBV infection, at least in B cells [95]. EVs from EBV transformed cells activate ERK and Akt signaling pathways in the recipient cell. They also contain high levels of the epidermal growth factor (EGF) receptor and can induce the expression of the EGF receptor in an EBV-negative epithelial cell line [95]. This promotion of growth signals has been speculated to have a role in the tumorigenic properties of EVs. In fact, EBV infection is relevant because the presence of oncoproteins and viral miRNAs in EVs released by EBV-infected cells, by regulating gene expression in the surrounding tissue, can alter immunosurveillance, thus impairing the destruction of the transformed cells by immune cells and favoring oncogenic transformation. Therefore, the inhibition of immune response may be linked not only to allowing the spreading of viral infection, but also to favoring oncogenic transformation.

EBV-infected cells can release host proteins, which can affect viral infectivity. Indeed, it was shown that EBV-infected cells contain the host protein galectin-9 [96]. This protein interacts with the Tim1 membrane receptor and induces apoptosis in T cells. Evidence was provided that vesicular galectin-9 can induce apoptosis of EBV-specific CD4^+^ cells, thus negatively regulating macrophage and T cell activation [97]. Interestingly, Raji cells infected by EBV release γ-interferon-inducible protein 16 (IFI16) [98]. This protein is involved in the recognition of the EBV genome by the innate immune response. It was hypothesized that its release outside the cell via EVs could contribute to escaping the recognition of the innate immune system. Indeed, the dual nature of EVs must be considered when the functional role of exosomes is investigated, as on one hand, they are signaling tools, but on the other hand, they are also used as a “garbage disposal” for unwanted molecules. Thus, they are not only involved in favoring viral spreading by downregulating immune response via diffusion of signals limiting immune reaction, but also contribute to the viral response down regulation, which eliminates host proteins that are relevant to prompt this response. Finally, another host protein that was retrieved in EVs released by EBV-infected cells is HIFα, which is also a pro-tumorigenic molecule [99]. This suggests that not only viral proteins released via EVs but also host proteins incorporated into EVs may be tumorigenic for the neighboring tissue.

As in the case of HIV, EBV-infected cells release EVs containing EBV nucleic acids, specifically viral miRNAs. EBV encodes 44 miRNAs distributed in three clusters, i.e., the BHRF1-cluster, the BART-1 cluster, and the BART-2 cluster, which target the cell and viral miRNAs to evade the immune system [100]. BHRF1 was demonstrated to repress the expression of the IFN-inducible T-cell attracting chemokine, CXCL11 [101]. Another EBV-encoded miRNA, miR-BART15, was shown to regulate the NLRP3 inflammasome and IL-1β production [102]. This miRNA also promotes cell apoptosis by targeting the apoptosis inhibitor BRUCE and TAX1BP1 [103,104]. The EV-mediated transfer of EBV miRNAs into recipient cells has been demonstrated by a few studies [95,105]. Pegtel et al. [25] reported the presence into EVs of the viral miRNA BHRF 1, which suppressed CXCL11. As this cytokine is involved in antiviral activity, the downregulation of its expression diminishes the antiviral response of the host cells. The miR-BART15-3p was shown to be highly enriched in EVs from an EBV-positive gastric cancer cell line, thereby inducing apoptosis in target cells, including immune system cells [103]. Thus, vesicular viral miRNAs appear to be involved in preventing an antiviral response, up to the induction of the death of the immune cells. It is important to note that miRNAs are sorted in a preferential manner into EVs, i.e., only a subset of miRNAs produced within a cell are retrieved within EVs. Even if it is known that there are several potential ways to sort miRNAs into vesicles [106], the mechanisms underlying viral the packaging of miRNAs into EVs have not been fully elucidated and may be virus-dependent, so we expect to gain further information on this aspect for different viruses in the future. EBV also actively expresses two non-protein coding RNAs (EBER1 and EBER2) [107]. EBERs contribute to viral carcinogenesis by blocking apoptosis and have been detected in EVs purified from the EBV-infected cell lines [108].

Besides EBV, other herpesviruses have been investigated, suggesting interesting mechanisms of infection spreading. Kaposi’s sarcoma-associated herpesvirus (KSHV) is a gamma herpesvirus like EBV. In contrast with EBV, KSHV -infected cells did not reveal the presence of viral proteins in EVs [109,110]. Instead, the packaging of host factors into EVs was reported. These EVs released from KSHV -infected cells could spread KSHV infection, mostly because the packaged host factors impaired the host’s immune response. In fact, it was shown that a KSHV infection can reprogram the metabolism of infected B cells toward glycolytic metabolism. EVs released from KSHV -infected cells are enriched with host proteins involved in glycolytic metabolism, such as lactate dehydrogenase, suggesting that these enzymes can be delivered via EVs and affect cell metabolism in recipient cells [109], thus contributing to viral persistence. KSHV latency also requires the downregulation of innate immune response. It was shown that cleaved IL-1 and IFI16 are released from infected cells within small EVs. This allows the extracellular elimination of host factors potentially relevant to initiate the innate immune response [110]. As for EBV, EVs released from KSHV infected cells may package KSHV-derived miRNA, which could play a role in the development of KSHV-associated malignancies, such as Primary Effusion Lymphoma (PEL) [111].

Other studies on Cytomegalovirus (CMV) and Herpes simplex virus 1 (HSV-1) have indicated a that EVs may have a role in increasing viral infectivity via elimination of the host protein relevant for antiviral response. CMV infection can increase the release of proteins that are necessary for virus uptake within EVs; these proteins include the lectin, DC-SIGN (dendritic cell-specific intercellular adhesion molecule-3 grabbing non-integrin). These vesicles were shown to mediate the CMV infection of myeloid DCs, increasing their infectivity [112]. HSV-1 proteins remodel the content of EVs released from infected cells. Specifically, the viral glycoprotein B attenuates the expression of HLA-DR at the cell surface by recruiting it to MVBs for exosome-mediated secretion [113]. HSV-1 encodes 27 mature miRNA sequences, many of which are involved in latency regulation [114]. EVs from HSV-1 infected cells have also been shown to contain viral mRNA and miRNA [115,116]. Moreover, two viral miRNAs (miR-H28 and miR-H29) that are produced late during HSV-1 infection have been exported in exosomes. When these miRNAs are ectopically expressed in human cells before infection, they reduce the accumulation of viral mRNAs and proteins. In addition, they are more abundant in cells where latent reactivation occurs. This evidence indicates that HSV-1 utilizes strategies to regulate its own expression to maximize its spread [116].

Finally, an important mechanism implicating a role for EVs in viral infectivity has been found while investigating Hepatitis C virus (HCV) biogenesis. HCV is a single strand RNA genome virus belonging to *Flaviviridae*. Although once thought to be assembled in the cytoplasm, at the level of ER, and released via the secretory pathway, it has been now recognized that this virus uses the cellular exosomal pathway for assembly and release [117]. This function has also been indicated by many other studies, which reported that HCV envelope proteins E1 and E2 and viral RNA are incorporated within exosomes. It has been also shown that, in addition to full viral particles, these exosomes can transmit HCV infection in a manner that can be compared to free viral particles [118]. As mentioned above, more technical development is needed to reliably separate viral particles from viral defective particles, exosomes enriched with viral elements and host exosomes. In order to avoid the problem of separation that can make it difficult to assign the ability to infect cells to exosomes, and not to co-separated virions, in a study by Longatti et al. [119], exosomes were isolated from a cell line lacking structural proteins and were, therefore, unable to produce virions. Even in this case, exosomes have shown the ability to infect cells. This evidence underlines that, at least for HCV, exosomes may be fully involved in the infection of recipient cells, which are responsible in the first grade of virus transmission. Further, exosome-mediated infection has the advantage of masking the HCV virus with a host envelope, further contributing to viral spread by two mechanisms: preventing an immune response because of the presence of host antigens and allowing infection via receptors that are different from viral receptors [120]. Finally, additional nucleic acid host components have been retrieved in exosomes released by HCV infected cells, which are involved in prompting viral replication. In particular, the Ago2 protein and miR-122 have been shown to enhance HCV replication via binding to the 5′UTR of HCV RNA [120,121].

### 3.2. Role of EVs in Eliciting Antiviral Response

The role of EVs in viral infection is not only limited to promoting viral spread, as different mechanisms can be activated by the EVs released by infected cells, thereby prompting an immune response against viruses. The most important mechanism, and first to be identified, is the spreading of viral antigens via EVs, in order to elicit an adaptive immune response against viruses. One of the best known examples has been given in a study on EBV [25,89], which demonstrated the B cell stimulatory capacity of EVs released by an EBV-infected B cell, thereby providing evidence that EVs influence B cell development in healthy EBV carriers with implications, for example, for allergy or autoimmune disease development [122].

However, the role of EVs in eliciting an antiviral response is not only limited to their role as delivery vehicles for viral antigens. In fact, additional mechanisms have been shown, all converging towards the stimulation of an immune response against viruses. In fact, EVs can transfer cytosolic proteins involved in antiviral responses. In the case of HIV, it has been demonstrated that EVs released by infected cells transport a host antiviral protein, APOBEC3G. This protein is a cytidine deaminase that inhibits viral replication by creating G to A mutations in the translated viral DNA. Although this host protein can be packed within virions, this process is limited because it is counteracted by the viral protein Vif. However, Vif is not packaged within EVs and, therefore, APOBEC3G packaged within EVs is much more effective [123]. Additional soluble host factors vehiculated via EVs can also contribute to the antiviral response against HIV. Cyclic GAMP, which is produced by cGAMP synthase, triggers an antiviral response mediated by interferon in infected cells and can be vehiculated by EVs (and possibly by incomplete or defective virions). Therefore, this finding indicates that defective particles can contribute to antiviral responses and not only to viral spreading, as in the case of HCV mentioned above [124,125].

In the case of EBV infection, it was reported that EBV-infected Raji cells release EVs containing deoxyuridine triphosphatase (dUTPase) [126] at sufficient levels to induce NF-kB activation and cytokine secretion in primary DCs and peripheral mononuclear blood cells (PBMCs), thereby suggesting that EBV-encoded dUTPase may act as an intercellular signaling molecule capable of modulating the cellular microenvironment, thereby stimulating an immune response. Therefore, EBV-encoded dUTPase may be important in the pathophysiology of diseases correlated with EBV infection, such as autoimmune diseases. Furthermore, it has been shown that EVs from an EBV-transformed B cell line preferentially target B cells through the interaction of the EBV-encoded glycoprotein gp350 with the CD21 receptor. Blocking EV uptake by targeting this interaction strongly inhibited EBV infection in B cells isolated from umbilical cord blood, suggesting a protective role for EVs in regulating EBV spreading [127]. EVs released by a Burkitt’s lymphoma cell line have been shown to be internalized by isolated B cells, leading to their proliferation, the induction of activation-induced cytidine deaminase, and the production of circle and germline transcripts for IgG1 [122]. Altogether, these results suggest that exosomes released from an EBV-infected B cell have a stimulatory capacity and interfere with the fate of the human B cell’s response.

IFI16 is considered to be an element that promotes latency by an innate immune response down modulation, which is responsible for the viral persistence for different herpesvirus, such as the EBV and KSHV, as mentioned in the previous section. However, in the case of CMV, the production of IFI16 acts as a restriction factor for CMV replication [128]. Further, it was demonstrated that upon CMV infection, endothelial cells release EVs carrying glycoprotein B. This protein could indirectly activate CD4^+^ cells, thereby inducing an adaptive immune response and helping to maintain a pool of T cells specific for the CMVs implicated in the control of CMV infection [129]. Studies on the exosomes released from HSV1-infected cells also showed the incorporation of the host product Stimulator of INF genes (STING) protein into vesicles. Together with viral miRNAs packaged within vesicles, STING exerted a negative effect on viral spread and increased host cell survival [115].

EVs can also transfer nucleic acids involved in antiviral responses. In this manner, cells can be reached by viral infection signals, although they are not infected by the virus itself, because, for example, they are devoid of the correct receptors. Although most of the genetic material enclosed in virions encodes for viral proteins that are essential for virus replication, viruses and EVs have a similar ability to transfer RNAs that can be recognized as Pathogen Associated Molecular Patterns (PAMPs) by Pathogen Recognition Receptors (PRRs) in target cells. Fragments of the viral genome, as well as virus encoded small RNAs and host cell miRNAs, have been shown to trigger target cell PRRs. Although triggering of the PRR system results in complex responses, in some cases it may induce an increased activation status of these cells. For example, recipient cells sense latent EBV infection through the vesicular transfer of 5′ capped RNA. HCV-permissive cells trigger a viral response in nonpermissive plasmacytoid DCs (pDCs) by transferring HCV-RNA-containing vesicles from infected cells to pDCs [130]. Further, viral miRNA can also be transmitted via EVs. In the case of HIV, two viral miRNAs, vmiR88 and -99, trigger endosomal TLR8 and NFkB signaling, stimulating the release of TNFα by delivering EV to bystander macrophages [131], thereby prompting an immune response against HIV. In some cases, the antiviral response may also not be due to the transfer of viral mRNAs or miRNAs via EVs, but because of the transfer of host cell miRNAs via EVs. These miRNAs are produced by virus resistant cells and then transmit this resistance to other cells, thus prompting an antiviral response. This is the case of placental trophoblasts, which form the interface between the fetal and maternal environments and serve to limit the maternal-fetal spread of viruses. It has been shown that cultured primary human placental trophoblasts are highly resistant to infection by several viruses, such as HSV-1 and CMV, and confer this resistance to nonplacental recipient cells by the EV-mediated delivery of miRNAs [132]. In the case of another herpesvirus, KSHV, it was recently shown that the presence of mitochondrial DNA released by infected cells via exosomes is able to initiate an antiviral response [30].

## 4. Conclusions

Virus infected cells produce EVs, which are important mediators of antiviral responses, as well as vehicles that facilitate viral infection. EVs released by virus-infected cells act through a multiplicity of mechanisms that contribute to the spread of viral infection. EVs carry viral proteins or nucleic acids, which are involved in virus reactivation during latency, as was shown for HIV and EBV, in hampering antiviral response (HIV and EBV), or in broadening viral tropism by delivering virus receptors in cells that are normally devoid of them (HIV). On the other hand, studies have demonstrated that EVs are also involved in stimulating antiviral mechanisms. In fact, EVs can spread cell molecules able to elicit an immune response, such as host soluble proteins and nucleic acids recognized as PAMPs.

Consequently, it appears that the role of EVs in viral infection and transmission is far from being satisfactorily elucidated. Notably, it is not clear what cell conditions and virus types release EVs that favor or fight infection. One underlying reason is the separation of viral particles from EVs in virally infected cells. This is a complex procedure, which cannot be completely accomplished by the currently available EV purification methods (for example, differential centrifugation or size exclusion chromatography). In fact, these protocols do not allow us to distinctly separate fully infective virions from defective viral particles and EVs containing viral elements. Therefore, the apparently contradictory effects of EVs from infected cells (pro- infection or pro-resistance) may be partially due to these technical problems, which may lead to contradictory results. Elucidating these points will also represent a breakthrough for the therapeutic applications of EVs in the immunological field, in order to exploit EVs for antiviral strategies.

Indeed, it is possible to imagine the engineering of EVs that could package viral elements or cell factors selected for their immunostimulatory properties, to be used as vaccines or tolerogenic tools in autoimmune diseases. A few studies have illustrated the potential use of “manipulated” EVs containing viral elements for vaccination purposes. Anticoli et al. [133] reported an exosome-based vaccine platform able to elicit cytotoxic T lymphocyte immunity against the E7 protein of the Human Papilloma Virus (HPV). “Manipulated exosomes” were obtained via the intramuscular injection of a DNA vector expressing HPV-E7 fused at the C-terminus of the exosome-anchoring protein Nef, previously characterized by its high level of incorporation in exosomes. The development of pharmacological agents interfering with EV production could also, in the future, allow us to modulate the release of EVs, tuning their antiviral properties according to therapeutic necessity. There is in vitro evidence that inhibitors of neutral sphingomyelinase can be used to inhibit the ceramide-dependent pathway of exosome biogenesis and that high-throughput screening may identify selective inhibitors of exosome biogenesis and secretion [134]. Furthermore, drugs interfering with lysosomal acidification may affect the release of EVs and alter their uptake [8,135].
